# Clove Essential Oil Enhances Antioxidant Defenses and Reduces DNA Damage in a Cellular Model of Parkinson’s Disease

**DOI:** 10.1155/padi/4243787

**Published:** 2025-12-15

**Authors:** Dhouha Hamdi, Omar Ouachik, Ayhan Kocer, Lemlih Ouchchane, Chokri Messaoud, Aziz Hafidi

**Affiliations:** ^1^ Laboratory of Nanobiotechnology and Valorization of Medicinal Phyto Resources, University of Carthage, National Institute of Applied Science and Technology UR17ES22, Tunis, Tunisia; ^2^ Institut Pascal, ICCN, TGI, University of Clermont Auvergne, Clermont-Ferrand, France, univ-bpclermont.fr; ^3^ GReD, University of Clermont Auvergne, Clermont-Ferrand, France; ^4^ CNRS, SIGMA Clermont, Institut Pascal, Université Clermont Auvergne, Unité de Biostatistique-informatique Médicale, 63000, Clermont-Ferrand, France, clermont-universite.fr

**Keywords:** 6-OHDA, antioxidant, CAT, DNA damage, GPx1, GPx4, neuroprotection, SOD1

## Abstract

Oxidative stress is a major contributor to the pathogenesis of Parkinson’s disease, promoting neuronal degeneration through the production of excessive reactive oxygen species. In this context, natural products such as essential oils are attracting increasing attention for their potential to protect neurons. Clove essential oil (CEO), which is extracted from the flower buds of the plant Syzygium aromaticum, is renowned for its antioxidant activity. This study aimed to investigate the chemical composition, antioxidant properties, and protective effects of CEO on SH‐SY5Y cells, which are used as a cellular model of Parkinson’s disease. The CEO was obtained by hydrodistillation (yield: 15%) and is primarily composed of eugenol (84.49%) and acetyl‐eugenol (10.05%). Its antioxidant activity was confirmed via DPPH radical scavenging (IC_50_ = 0.081 ± 0.001 mg/mL) and iron chelation (110.32 ± 0.67 mg EDTA equivalent/g essential oil (EO)). qRT‐PCR, Western blot, and slot blot techniques demonstrated that CEO pretreatment at concentrations of 2.5, 5, and 10 μg/mL significantly reduced 6‐OHDA‐induced oxidative DNA damage and restored the gene and protein expression of key antioxidant enzymes (GPx1, GPx4, SOD1, and CAT). These results highlight the powerful antioxidant and neuroprotective properties of CEO, supporting its potential as a therapeutic agent for neurodegenerative disorders related to oxidative stress, such as Parkinson’s disease.

**Highlights**

• Clove essential oil (CEO) extracted from Syzygium aromaticum flower buds demonstrates significant antioxidant and neuroprotective properties.
• CEO is primarily composed of 84.488% eugenol (EG) and 10.05% acetyl‐EG, contributing to its bioactive profile.
• The antioxidant capacity of CEO was confirmed through DPPH free radical scavenging and iron chelation assays, showing strong efficacy.
• CEO pretreatment in SH‐SY5Y cells significantly protected against oxidative stress induced by 6‐OHDA neurotoxin.
• CEO reduced oxidative DNA damage and restored the expression of key antioxidant enzymes (GPx1, GPx4, SOD1, CAT).
• The study suggests CEO as a promising therapeutic agent for oxidative stress‐related neurodegenerative diseases, such as Parkinson’s disease.

## 1. Introduction

Oxidative stress arises from an imbalance between the production of reactive oxygen species (ROS) and a cell’s antioxidant defenses. Although ROS are a natural by‐product of cellular metabolism and play a role in signaling and homeostasis, their accumulation can damage DNA, proteins, and lipids [1, 2]. To counteract this, cells rely on antioxidant enzymes, such as superoxide dismutase (SOD), catalase (CAT), and glutathione peroxidases (GPx1 and GPx4), to neutralize ROS [3, 4]. GPx1 primarily detoxifies hydrogen peroxide, while GPx4 reduces lipid peroxides, thereby protecting against ferroptosis. These enzymes are essential for maintaining redox balance and preventing age‐related and neurodegenerative diseases [4].

Excessive oxidative stress can impair mitochondrial function and DNA repair mechanisms, which can contribute to the development of neurodegenerative disorders such as Parkinson’s disease (PD), Alzheimer’s disease (AD), and amyotrophic lateral sclerosis [5, 6]. In PD, for example, the antioxidant systems of dopaminergic neurons become dysregulated, resulting in mitochondrial DNA damage and progressive neuronal loss [7]. Therapeutic strategies based on antioxidants have shown promise in slowing neurodegeneration [8]. Natural compounds, particularly plant‐derived antioxidants, are considered promising candidates due to their efficacy, safety, and sustainability [9]. CEO, which is rich in phenolic compounds, has demonstrated antioxidant and neuroprotective properties in experimental models of AD and PD [10, 11].

This study investigates the neuroprotective potential of CEO against 6‐hydroxydopamine (6‐OHDA)‐induced oxidative stress in SH‐SY5Y human neuroblastoma cells, which are a well‐established in vitro model of PD. It is hypothesized that pretreatment with CEO may reduce oxidative damage, boost endogenous antioxidant defenses, and bolster neuronal resilience, thus highlighting its potential as a natural therapeutic agent for neurodegenerative disorders associated with oxidative stress.

## 2. Materials and Methods

### 2.1. Extraction and Analysis of Essential Oils (EOs)

The hydrodistillation of EO was performed using 100 g of clove flower buds purchased from the local market in Tunis, Tunisia, with a Clevenger‐type apparatus [12]. After a hydrodistillation period of 24 h, the obtained extracts were collected. The EOs were then separated from the hydrosol, devoid of any traces of water, and stored in opaque glass containers protected from light at a temperature of 4°C. The composition of the EO was determined using gas chromatography coupled with mass spectrometry (GC–MS). The chromatograph used was an Agilent 7890A, equipped with an HP‐5MS column (30 m × 0.25 mm; film thickness 0.25 μm), a split/splitless injector, and an Agilent 5975C inert mass selective detector (MSD). During the analysis, the oven temperature was gradually increased from 60°C to 240 °C at a rate of 4°C/min, followed by isothermal maintenance at 240°C for 10 min. The injector temperature was set to 250°C, and the interface temperature was 280°C. The ionization source temperature was 230°C, while the quadrupole temperature was maintained at 150°C. The ionization energy was set to 70 eV, and the mass scanning range varied from 50 to 550 amu at a rate of 2.91 scans/s. Helium was used as the carrier gas at a flow rate of 0.8 mL/min. For each sample, 1 μL of EO, previously diluted in hexane (1:100), was injected. The compounds in the EOs were identified by comparing their mass spectra with those recorded in the NIST08 and W8N08 databases. Identification was also confirmed by comparing the retention indices, determined by reference to a homologous series of n‐alkanes C9‐C20, with those described in the literature.

### 2.2. Evaluation of Antioxidant Activities

#### 2.2.1. 2,2‐Diphenyl‐1‐picrylhydrazyl (DPPH) Radical Scavenging Assay

DPPH is a stable synthetic radical in solution that exhibits a deep violet color in its oxidized state. In the presence of hydrogen donors (antioxidants), DPPH is reduced to 2,2‐diphenyl‐1‐picrylhydrazine, which is yellow in color. This discoloration corresponds to a decrease in absorbance at 517 nm, which is directly proportional to the radical scavenging capacity of the tested sample [13]. To assess the DPPH radical scavenging activity, we followed the method described by Brand‐Williams et al. [13]. Briefly, 50 μL of CEO (at different concentrations in methanol) was mixed with 1.95 mL of a 60 μM DPPH radical solution. After homogenization, the mixture was incubated in the dark for 30 min. The absorbance was then measured at 517 nm. The radical scavenging activity was calculated as the percentage of radical scavenging activity (I %) according to the following formula: I % = [(A0 ‐ A1)/A0] x100, where A0 represents the absorbance of the control reaction and A1 is the absorbance of the tested sample. Finally, the radical scavenging activity was expressed in terms of the IC50 value, defined as the concentration of the antioxidant required to neutralize 50% of the DPPH radicals present in the tested solution.

#### 2.2.2. Evaluation of Iron Chelation Capacity

This is a colorimetric assay based on the chelation of ferrous ions (Fe^2+^) by ferrozine. An intense magenta color appears when the complex forms, with the Fe^2+^–ferrozine complex exhibiting an absorbance at 562 nm. The ability to decolorize the complex (Fe/ferrozine) reflects the chelation capacity of the extracts, as described in the study by Stookey in 1970. The iron chelation activity of CEO was measured by the decrease in absorbance at 562 nm of the ferrous–ferrozine complex [14]. Five hundred microliters of different concentrations of EO in methanol were added to 500 μL of FeSO_4_ solution (0.125 mM) and incubated at room temperature for 5 min. The reaction was then initiated by adding 500 μL of ferrozine (0.3125 mM). The mixture was vigorously agitated and allowed to stand at room temperature for 10 min. The absorbance of the solution was measured at 562 nm against a methanol blank. The capacity to chelate the ferrous ion was calculated using the following formula: Chelation Effect (%) = [100 × (AC ‐ AS/AC)], where AC is the absorbance of the control and AS represents the absorbance of the tested sample. The results were expressed in EDTA equivalents per gram of EO (EDTA eq/g EO).

### 2.3. Study of Oxidative Stress Biomarkers

#### 2.3.1. Cell Preparation

In this experiment, cells were seeded in 6‐well plates using complete DMEM medium 1X (31885‐023, Gibco). The medium was supplemented with 1% of 100X vitamins (11120‐037, Gibco), 1% of 100X nonessential amino acids (11140‐035, Gibco), 10% heat‐inactivated fetal bovine serum (FBS) (F9665, Sigma), and a 100X penicillin–streptomycin solution (P0781, Sigma). Six groups of cells (SH‐SY5Y cell line, Sigma‐Aldrich, France) were analyzed: a positive control without treatment, a group of cells treated with 100 μM of 6‐OHDA for 24 h, and four additional groups pretreated for 1 hour with different concentrations of CEO (at 2,5; 5; 10; and 20 μg/mL), followed by treatment with 100 μM of 6‐OHDA. The CEO concentrations (2.5–20 μg/mL) were selected based on our previous cytotoxicity evaluation [15], in which these doses showed no toxicity in SH‐SY5Y cells while providing significant neuroprotection. After 24 h of treatment, the cells were detached, centrifuged for 5 min at 800 rpm, and then stored at −80°C for subsequent manipulations. For each culture group, cells were seeded and treated in triplicate (n = 3).

#### 2.3.2. DNA, RNA, and Proteins Extraction

The extraction of DNA, RNA, and proteins was performed simultaneously on the six groups of cells using a specific kit (AllPrep DNA/RNA/Protein Mini Kit (50)).

#### 2.3.3. DNA Damage Assessment

To evaluate the protective effect of CEO against oxidative DNA damage caused by 6‐OHDA, a slot blot analysis was conducted using the DNA damage antibody, 8‐OHdG (8‐hydroxy‐2′‐deoxyguanosine or 8‐hydroxyguanosine). For this analysis, DNA samples were diluted to 50 ng/mL. A nylon membrane (RPN203B) was secured using a vacuum transfer device, and samples were applied to the apparatus well. After the sample application, the membrane was crosslinked by UV irradiation at 125 mJoules. Subsequently, the membrane was blocked with a saturation solution (1X PBS, 10% milk, 1% BSA, 0.1% Tween 20) overnight at 4 °C. Next, the membrane was incubated with the primary antibody 8‐OHdG (1:1000) diluted in the saturation solution for 1 h at 4 °C with agitation. After incubation, the membrane was washed three times for 5 min each with PBS‐T (PBS + 0.1% Tween 20). The secondary antibody conjugated to HRP was prepared in the saturation solution (1:5000), and the membrane was incubated for 1.5 h at room temperature. The membrane was washed again three times with PBS‐T for 5 min each time. Detection was performed using ECL substrate, and the chemiluminescence signals were captured using a Bio‐Rad machine to visualize DNA damage. Finally, the bands were analyzed using Bio‐Rad’s Image Lab software. All experiments were independently performed in triplicate (*n* = 3).

#### 2.3.4. RT‐PCR

The amplification reaction was performed in a total volume of 25 μL, containing 2 μL of RNA, 0.5 μL of each primer, sterile distilled water, 5 μL of buffer, 5 μL of dNTPs, 0.5 μL of RNase inhibitor, and 0.5 μL of reverse transcriptase. The amplification reaction was conducted in a thermal cycler. The PCR program included an initial step of 5 min at 70°C. The RNA, primers, and water mixture were placed on ice, followed by the addition of the reaction mixture. The tubes were then reinserted into the thermal cycler for 1 h at 37°C. Finally, a postelongation step at 72°C was performed for 10 min. The PCR product was subsequently stored at 4°C.

#### 2.3.5. qRT‐PCR: Expression of GPx1, GPx4, SOD1, and CAT Genes

The expression of antioxidant system genes was analyzed using quantitative PCR on the RT‐PCR product. Quantitative RT‐PCR was conducted as described by Batista et al. [16], utilizing the Platinum SYBR Green qPCR SuperMix‐UDG (Invitrogen) and the Roche Light Cycler PCR system. The melting temperatures of the amplicons ranged from 60°C to 62 °C. The primers used were as follows (Table 1). Each experimental condition was tested in three independent replicates (*n* = 3). β‐Actin was used as a single reference gene due to its stable expression across all experimental conditions.

**Table 1:** Primer sequences.

Primer	Forward	Reverse
GPx1	5′‐CAG ATG AAC GAG CTG CAG CG‐3′	5′‐TCG GTC ATA AGC GCG GT G GC‐3′
GPx4	5′‐TCC GCC AAG GAC ATC GACGG‐3′	5′‐TCC CGA ACT GGT TAC ACG GG‐3′
CAT	5′‐GAT CCT GAC TAT GGC ATC CG‐3′	5′‐CTG GGA TGA GAG GGT AGT CC‐3′
SOD1	5′‐CTG TAC CAG TGC AGG TCC TC‐3′	5′‐AAT GAT GCA ATG GTC TCC TG‐3′
β‐Actin	5′‐GGA CTT CGA GCA AGA GAT GG‐3′	5′‐AGC ACT GTG TTG GCG TAC AG‐3′

#### 2.3.6. Western Blot: Expression of GPx1, GPx4, SOD1, and CAT Proteins

To assess the expression of GPx1, GPx4, SOD1, and CAT proteins in order to determine the effect of 6‐OHDA and CEO on the oxidative stress regulatory system, Western blotting was performed using specific primary antibodies for these proteins. Experiments were independently repeated in triplicate (*n* = 3). Samples were diluted in ALO buffer, heated at 95 °C for 5 min, then vortexed and centrifuged. The stain‐free precast gel was prepared by removing the protective film and combs, and the wells were checked with a pipette. Five μL of molecular weight marker and 15 μL of sample (containing 20 μg of total protein, adjusted to equal concentration across all lanes) were loaded per well, depending on the gel. Proteins were migrated at 70 V for 15–20 min, then at 200 V for 30–40 min. After migration, the transfer was carried out using the Trans‐Blot Turbo transfer kit (Bio‐Rad) with a nitrocellulose membrane. The transfer buffer (1X) was prepared, and buffer papers were soaked before assembling them with the gel and membrane in the Trans‐Blot Turbo transfer system (Bio‐Rad), ensuring even spreading to avoid air bubbles. The transfer was performed at 2.5 A, 25 W for 7 min. The membrane was stained with Ponceau red to verify migration, then rinsed and blocked with TBST containing 5% milk for at least 1 h at room temperature. Primary antibodies (GPx4 Ab125066; GPx1; CAT 14097S; SOD1 VL315171 at 1/1000; and β‐actin at 1/10000) were prepared in TBST with 5% milk, and membranes were incubated overnight at 4 °C. The primary antibody solution was recovered and stored at −20 °C. Membranes were washed three times with 1X TBST for 10 min each. Secondary antibodies were prepared in TBST with 5% milk and incubated for 1 h at room temperature; then, membranes were washed again three times with 1X TBST. Finally, detection was carried out using the Clarity Western ECL Substrate kit, and the Bio‐Rad device was used to visualize and photograph the proteins. The Bio‐Rad imaging system was used to visualize signal intensities, which were then analyzed using Image Lab software. β‐Actin was used as a loading control, and the band intensities were normalized accordingly. Care was taken to avoid signal saturation, and only bands within the linear range of detection were quantified.

### 2.4. Statistical Analysis

Data are presented as the mean ± standard error of the mean (SEM) from at least three independent experiments. Statistical differences between groups were evaluated using a one‐way analysis of variance (ANOVA), followed by a Tukey’s honestly significant difference (HSD) post hoc test for multiple comparisons. A *p*‐value of less than 0.05 was considered statistically significant. All analyses were performed using SAS software, Version 9.4 (SAS Institute Inc., Cary, NC, USA).

## 3. Results

### 3.1. EO Analysis

The yield, calculated on a dry weight basis, showed that clove flower buds are very rich in EO, with a yield of 15% obtained after 24 h of hydrodistillation.

The terpenic composition of CEO was analyzed by GC–MS. This EO is mainly composed of 7 compounds (Table 2). CEO flower buds are characterized by a chemical composition largely dominated by phenols, accounting for 94.538% of the total composition. The predominant compound is EG with a concentration of 84.488%, followed by acetyl‐EG with 10.05%. Sesquiterpenes, although present in smaller quantities, represent 5.461% of the total CEO composition. Among the main sesquiterpenes identified, caryophyllene is the most abundant with 3.546% of the total. Other sesquiterpenes such as humulene (0.473%), caryophyllene oxide (0.994%), α‐copaene (0.352%), and humulene oxide II (0.096%) are also present but at very low levels.

**Table 2:** Chemical composition (%) of clove flower bud essential oil.

Chemical compound	RI	%
Eugenol	1292	84.488
α‐Copaene	1303	0.352
Caryophyllene	1332	3.546
Humulene	1354	0.473
Acetyl‐eugenol	1398	10.05
Oxyde de caryophyllene	1438	0.994
Oxyde d’humulene II	1454	0.096
Total identified (%)		100
Phenols (%)		94.538
Sesquiterpenes (%)		5.461

Abbreviation: RI, retention indices relative to n‐alkanes on an HP‐5MS column.

### 3.2. Antioxidant Activity

Two tests, the DPPH radical scavenging assay and the iron chelation capacity assay, were used to evaluate the antioxidant properties of CEO. The CEO from the flower buds of *Syzygium aromaticum* showed significant antioxidant activity with an IC50 value of 0.081 ± 0.001 mg/mL for DPPH radical reduction, which was comparable to that of Trolox (IC_50_ = 0.076 ± 0.004 mg/mL) used as a positive control. In addition, an iron chelating capacity of 110.321 ± 0.67 mg EDTA eq/g EO was observed, further confirming the antioxidant efficacy of EO from *S. aromaticum* flower buds.

### 3.3. Protective Effect of CEO Against Oxidative Stress Induced by 6‐OHDA

6‐OHDA at a concentration of 100 μM was used because it has previously been shown to be significantly neurotoxic to SH‐SY5Y cells, resulting in approximately 59% cell death [15]. One hour of CEO pretreatment of SH‐SY5Y cultures at various concentrations (2.5, 5, 10, and 20 μg/mL) significantly protected these cells from 6‐OHDA‐induced cytotoxicity [15]. This neuroprotective effect was attributed to the antioxidant activity of CEO. To further investigate this effect, DNA damage assays were performed in addition to expression analysis of genes and proteins related to oxidative stress, including GPx1, GPx4, CAT, and SOD1. Six groups of cell cultures were included in the study (Table 3).

**Table 3:** Groups of cell cultures.

Groups	Pretreatment (CEO μg/mL)	Treatment (6‐OHDA [100 μM])
Control	—	—
Negative control	—	+
G‐2.5	2.5	+
G‐5	5	+
G‐10	10	+
G‐20	20	+

### 3.4. Protective Effects of CEO on 6‐OHDA‐Induced DNA Damage

Oxidative DNA damage in SH‐SY5Y cells was assessed by measuring the expression levels of 8‐OHdG using the slot blot technique. Six groups of cells were labeled with 8‐OHdG to evaluate the efficacy of CEO in preventing oxidative DNA damage. Statistical analysis was performed on results obtained from three biological replicates. Exposure of SH‐SY5Y cells to 100 μM 6‐OHDA for 24 h resulted in a significant increase in DNA oxidation (*p* < 0.05) compared to control cultures. Pretreatment with CEO at concentrations of 2.5, 5, and 10 μg/mL for one hour prior to exposure to 100 μM 6‐OHDA (24 h) significantly reduced oxidative DNA damage (*p* < 0.05) compared to cells treated with 6‐OHDA alone (Figure 1). No significant differences were detected between the untreated control group and the groups that were pretreated with CEO at concentrations of 2.5 and 5 μg/mL.

Figure 1Protective effects of 1 h pretreatment with CEO on oxidative DNA damage in SH‐SY5Y cells induced by 100 μM 6‐OHDA for 24 h. Levels of 8‐OHdG are measured as the intensity of labeling on the membrane using the slot blot technique. Values are expressed as a percentage relative to the control (set at 100%) and presented as mean ± SEM (a) from three independent experiments. Statistical analysis was performed using one‐way analysis of variance (ANOVA) followed by the THSD test. The labeling intensity in the 6‐OHDA‐treated group is significantly higher than in the control group (^∗∗∗^ *p* < 0.05). This labeling is significantly reduced (*p* < 0.05) by treatment with CEO at concentrations of 2.5, 5, and 10 μg/mL. Representative slot blot membrane images are shown to illustrate the differences in 8‐OHdG signal intensity between groups (b). A 3D surface plot generated using Bio‐Rad’s ImageLab software provides a visual representation of signal distribution and intensity across the membrane (c).

**Figure:**
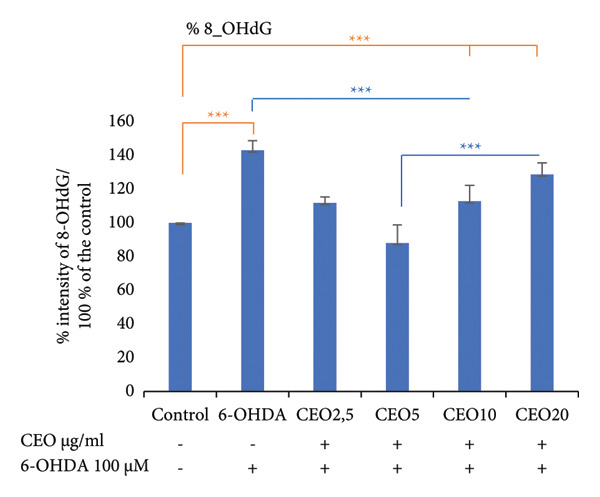
(a)

**Figure:**
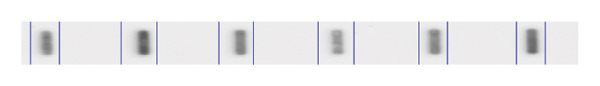
(b)

**Figure:**
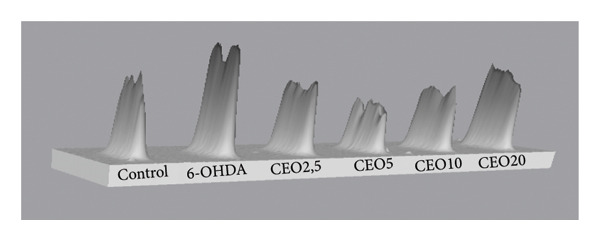
(c)

Notably, the 5 μg/mL concentration produced the most significant protective effect, which is consistent with the findings of the cell viability assay [15].

### 3.5. qRT‐PCR: Expression of Oxidative Stress Genes (GPx1, GPx4, CAT, SOD1)

The effect of 6‐OHDA and CEO on the key antioxidant genes expression of GPx1, GPx4, CAT, and SOD1 was investigated using qRT‐PCR. As illustrated in Figure 2, treatment with 6‐OHDA led to a significant decrease (*p* < 0.05) in the expression of these oxidative stress‐related genes compared to untreated control cultures. CEO treatment at all concentrations significantly restored gene expression levels in 6‐OHDA‐treated cell cultures, with 5 μg/mL demonstrating the most effective response. When compared to control cell cultures, this concentration resulted in a significant (*p* < 0.05) increased the expression of GPx1 (Figure 2(a); 55.22% compared to control cell cultures) and GPx4 (Figure 2 B; 69.14% compared to control cell cultures) and nearly a complete recovery of SOD1 (Figure 2(c); 94.79%) and CAT (Figure 2(d); 80.45%). The highest CEO concentration (20 µg/mL) did not significantly affect all gene expressions.

Figure 2Effect of CEO on the relative expression of oxidative stress biomarker genes (GPx1, GPx4, SOD1, and CAT) at the RNA level in SH‐SY5Y cell cultures induced by the neurotoxin 6‐OHDA (100 μM for 24 h). Gene expression levels were measured by quantitative RT‐PCR. Data were normalized using the housekeeping gene β‐actin as an internal control and are presented as percentages relative to the control, which was set at 100%. Each value represents the mean (±SEM) of three independent experiments. Statistical analysis was performed using one‐way analysis of variance (ANOVA) followed by the THSD test. Exposure to 6‐OHDA resulted in a significant inhibition (*p* < 0.05) of the expression of the genes (GPx1, GPx4, SOD1, and CAT). Pretreatment with different concentrations of CEO for 1 hour significantly restored the expression of these genes in 6‐OHDA‐treated cell cultures. Although the highest concentration of CEO showed an increase in gene expression, this increase was not significant.

**Figure:**
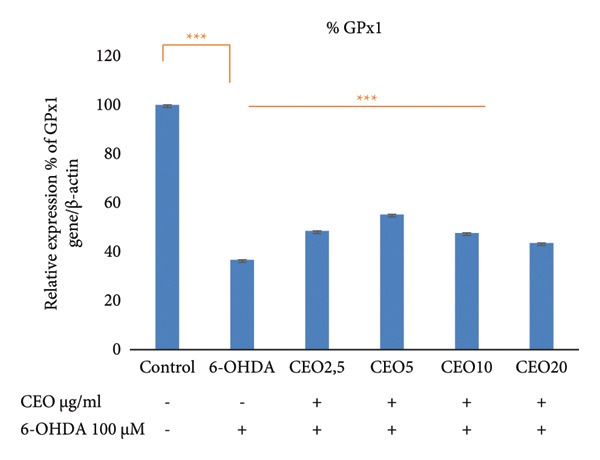
(a)

**Figure:**
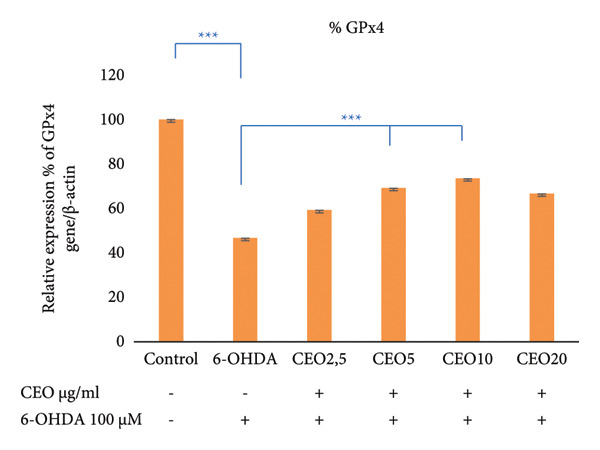
(b)

**Figure:**
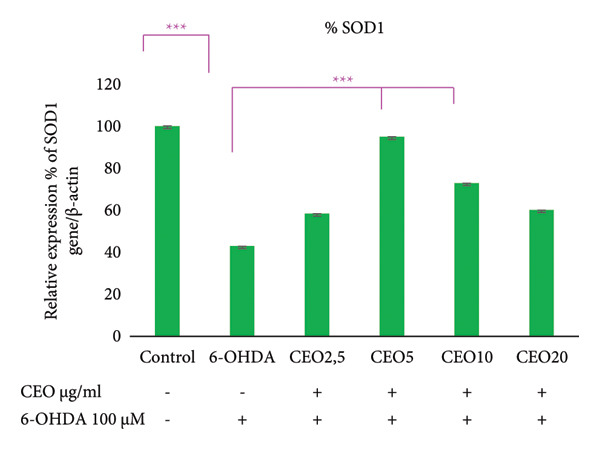
(c)

**Figure:**
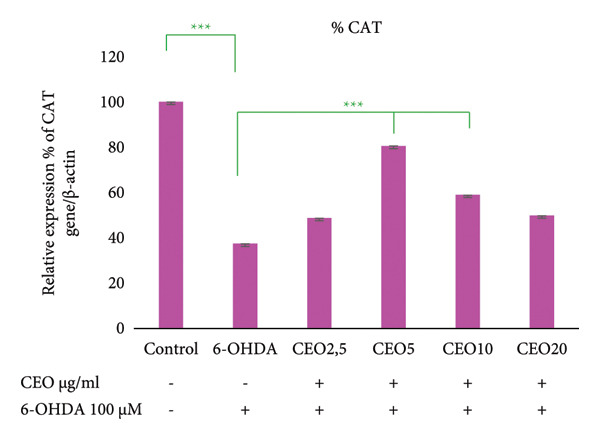
(d)

### 3.6. Western Blot: Relative Expressions of Oxidative Stress Proteins (GPx1, GPx4, CAT, SOD1)

To further assess oxidative stress in the cells, the expression of oxidative stress biomarkers (GPx1, GPx4, SOD1, and CAT) was measured at the protein level by Western blot (Figure 3). The intensity of each band was normalized against the corresponding β‐actin band. Triplicate experiments were performed to ensure reproducibility and reliability. Protein expression levels are reported as a percentage relative to the control group (set to 100%). Exposure of SH‐SY5Y cells to 100 μM 6‐OHDA for 24 h resulted in a significant (*p* < 0.05) decrease in the expression of all four antioxidant proteins of 59.55%, 59.29%, 53.25%, and 44.17% for GPx1 (Figure 3(a)), GPx4 (Figure 3(b)), SOD1 (Figure 3(c)), and CAT (Figure 3(d)), respectively. Pretreatment with different doses of CEO for 1 hour significantly increased the expression of GPx1, GPx4, SOD1, and CAT proteins in SH‐SY5Y cell cultures compared to 6‐OHDA‐treated cultures. GPx1 protein expression levels increased considerably (*p* < 0.05) at dosages of 2.5, 5, 10, and 20 μg/mL, reaching 68.20%, 82.29%, 76.03%, and 70.13% of the level of 6‐OHDA‐untreated control cell cultures, respectively. Similarly, the relative expression of GPx4 and SOD1 proteins showed improvement at varied CEO concentrations of 2.5, 5, and 10 μg/mL, reaching 74.74%, 81.48%, and 68.33% for GPx4 and 65.81%, 77.88%, and 62.13% for SOD1 compared to 6‐OHDA‐untreated cultures, respectively. CAT protein expression increased significantly (*p* < 0.05) at CEO doses of 2.5, 5, 10, and 20 μg/mL, reaching 68.31%, 90.73%, 79.88%, and 75.83% of the 6‐OHDA‐untreated cell cultures. A high dose of 20 μg/mL of CEO did not significantly increase the expression of GPx4 and SOD1 proteins.

Figure 3Effect of various CEO concentrations on the expression of oxidative stress Biomarker proteins (GPx1, GPx4, SOD1, and CAT) in SH‐SY5Y cells treated with 100 μM 6‐OHDA. The relative protein expression levels, normalized to β‐actin, for GPx1 (a), GPx4 (b), SOD1 (c), and CAT (d) are expressed as a percentage compared to the control group (set at 100%). The data are presented as mean ± SEM from three independent experiments. One‐way analysis of variance (ANOVA) followed by Tukey’s HSD test revealed a significant decrease in protein expression in the 6‐OHDA‐induced cell group (^∗∗∗^ *p* < 0.05) compared to the untreated control groups. In the presence of CEO at different doses, an improvement in this protein expression was observed. (e) Representative Western blot images showing the expression of GPx1, GPx4, SOD1, CAT, and β‐actin under the different treatment conditions.

**Figure:**
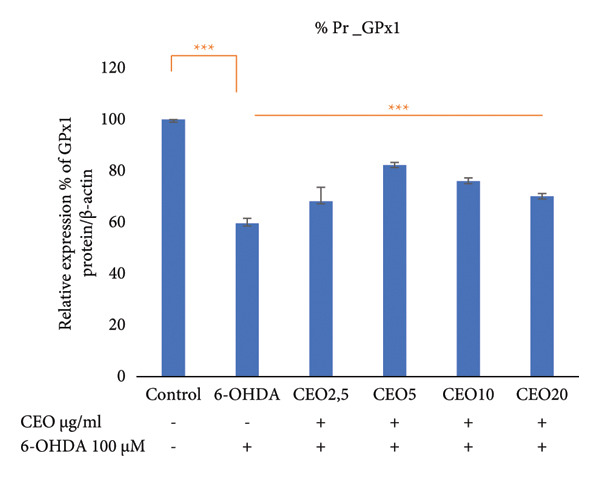
(a)

**Figure:**
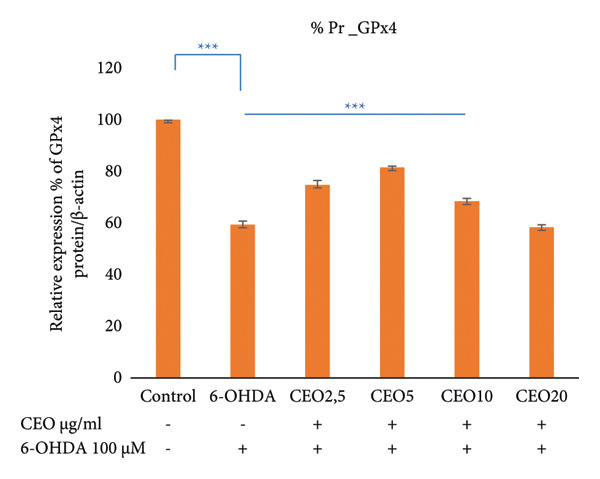
(b)

**Figure:**
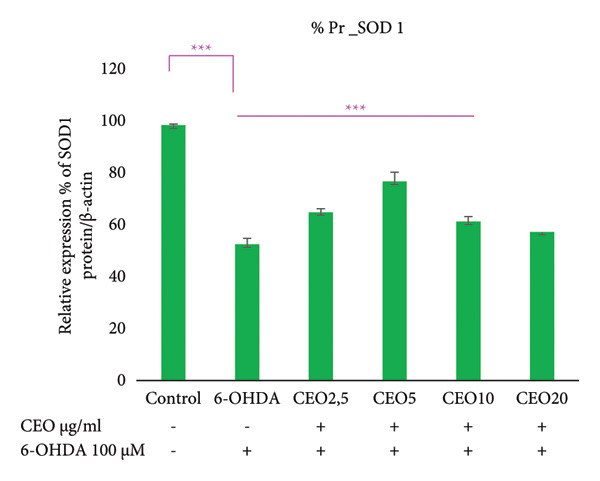
(c)

**Figure:**
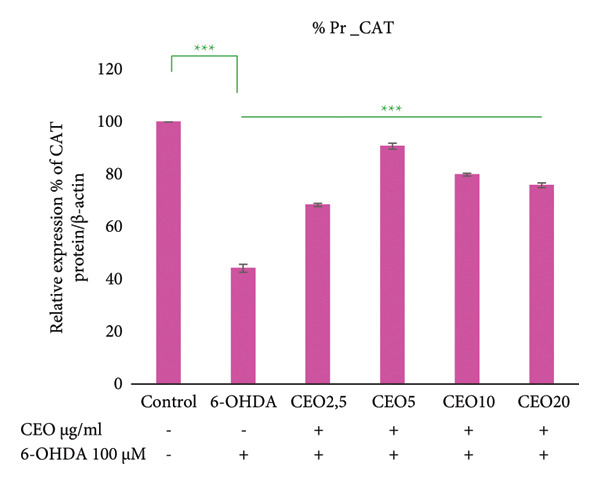
(d)

**Figure:**
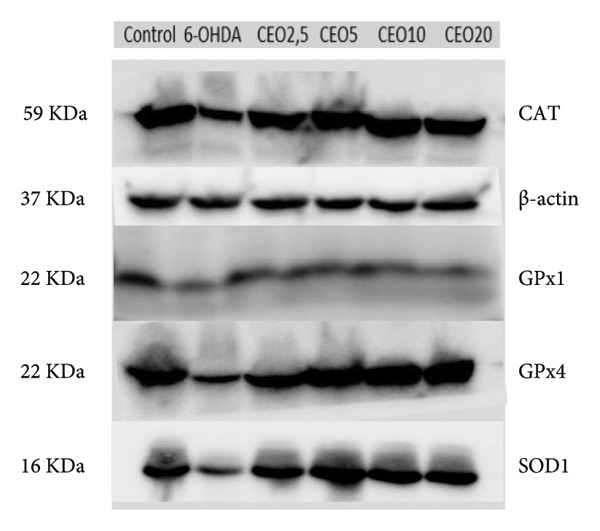
(e)

## 4. Discussion

There is a growing interest in using natural plant chemicals to slow the progression of PD [17]. CEO has been demonstrated to protect substantia nigra dopaminergic neurons from 6‐OHDA‐induced cell death while also improving motor performance in 6‐OHDA‐treated rats [15]. Because CEO has a neuroprotective effect against 6‐OHDA, the current work sought to evaluate the potential effects of this oil on the expression of important oxidative stress enzymes (GPx1, GPx4, CAT, SOD1) and the associated 6‐OHDA‐induced DNA damage (8‐OHdG). The current study demonstrated that: (1) The primary components of CEO are EG and acetyl‐EG. (2) CEO has significant antioxidant activity, as demonstrated by two distinct experiments (DPPH and iron chelation tests). (3) CEO protected against 6‐OHDA by upregulating the expression of various oxidative stress enzymes (GPx1, GPx4, CAT, and SOD1). (4) CEO reduced 6‐OHDA‐induced DNA damage, as evidenced by the low expression of 8‐OHdG in SH‐SY5Y cells.

### 4.1. Analysis of CEO

The utilization of EOs is heavily dependent on the yield of aromatic plants, which impacts the amount of EO collected during the extraction process. *Syzygium aromaticum* is well recognized for its unopened flower buds, which contain a high concentration of oil [18]. In this investigation, clove flower buds were shown to be high in EO. Hydrodistillation of these buds took 24 h and yielded 15%. The 24‐h hydrodistillation could potentially influence the stability of volatile compounds; however, the high proportion of EG and acetyl‐EG observed here suggests that prolonged distillation did not markedly degrade the oil’s major constituents. Similar yields and profiles have been reported for long extraction durations [19], confirming that extended hydrodistillation can still produce chemically stable and bioactive oils. This finding is consistent with other research that has shown yields of 11.5% [20], 10.54% [21], 11.6% [22], and 12.96%–16.73% [19]. However, our findings differ from those published in previous investigations [23, 24], in which yields ranged from 0.18% to 7.6%. These discrepancies in oil yield from buds could be attributed to the flowering stage versus the early bud stage, as well as the age and origin of the clove [19].

CEO’s terpene composition was studied by GC–MS, revealing a preponderance of EG (84.49%), acetyl‐EG (10.05%), and β‐caryophyllene (3.55%). Several studies have consistently identified these chemicals as the primary components of *Syzygium aromaticum* EO, albeit in varying quantities [19, 22, 24–28].

Previous research indicates that a variety of factors determine CEO’s chemical composition, including ambient circumstances, genotype, geographical origin, harvest time, drying process, temperature, and storage duration. The quality of CEO is principally determined by EG and eugenyl acetate, while its aroma and distinctive properties are shaped by hydrocarbon concentration [19]. Additionally, CEO derived from flowering buds has a higher EG concentration and a more complex chemical profile [19].

### 4.2. Antioxidant Activity

In the present study, CEO exhibited strong antioxidant activity as measured by DPPH and iron chelation assays. The main component of CEO, EG, is renowned for its powerful antioxidant capabilities [29, 30]. Previous studies have demonstrated that EG has higher antioxidant activity than synthetic chemicals, such as BHA [31]. Our study’s IC50 (DPPH) value of 0.081 ± 0.001 mg/mL is consistent with other findings, confirming the potent antioxidant activity of EG over a range of IC50 values: 0.36 μL/mL [32], 4.5 μg/mL [28], 23.17 μg/mL [24], 396.3 μg/mL [33], and 380 μg/mL (Viuda‐Martos et al.). CEO’s remarkable antioxidant activity has been linked to its high levels of EG and eugenyl acetate [34], as well as synergistic interactions between EG and other minor components in the oil [24]. EG’s outstanding radical scavenging efficacy might be explained by three main modes of action: hydrogen donation followed by delocalization of the para‐substituted group, dimerization between two phenoxyl radicals, and DPPH complexation with an aryl radical [24]. Saeed and Shahwar [28] demonstrated that EG’s antioxidant activity against ROS and lipid peroxidation (LPO) is due to the presence of a phenolic group capable of scavenging an alkyl radical by donating a hydrogen atom from the phenolic hydroxyl group. CEO has strong antioxidant properties and can be exploited as a readily available source of natural antioxidants in pharmaceutical applications [35].

### 4.3. Protection of DNA and the Antioxidant System With CEO in SH‐SY5Y Cells

Our most recent study found that CEO protects SH‐SY5Y cells from 6‐OHDA‐induced cytotoxicity [15]. The current study examined how CEO affected the expression of numerous enzymes involved in oxidative stress (GPx1, GPx4, CAT, SOD1) and DNA damage (the presence of 8‐OHdG).

Oxidative stress generates ROS, which are known to cause DNA damage, particularly in mitochondria [36, 37]. 8‐OHdG is the most widely used biomarker of ROS‐induced DNA damage [38]. Elevated levels of 8‐OHdG have also been observed in the substantia nigra of PD’s patients [39]. The addition of 6‐OHDA to SH‐SY5Y cell cultures increased DNA oxidation [40] and 8‐OHdG expression [41]. The present study’s rise in 8‐OHdG expression is consistent with the findings of Pradhan et al. [41]. High doses of 8‐OHdG have been shown to trigger apoptosis in SH‐SY5Y cells [42]. Monoamine oxidase (MAO) deaminates 6‐OHDA, which generates more ROS. In fact, accumulation of 6‐OHDA in mitochondria inhibits respiratory chain activity by blocking complex I, leading to indirect oxidative stress [43]. This biochemical cascade causes oxidative stress that exceeds the capacity of detoxification systems, ultimately leading to apoptotic cell death [44]. Pretreatment with CEO significantly reduced DNA damage in SH‐SY5Y cells (*p* < 0.05) as evaluated by 8‐OHdG expression. This reduction of DNA damage is most likely owing to its iron chelating activity. Iron chelators prevent 8‐OHdG production and 6‐OHDA‐induced apoptosis [42]. Treatment with EG, the primary component of CEO, significantly decreased nitric oxide (NO) and 8‐OHdg levels [45]. CEO and EG had comparable protective effects against 6‐OHDA‐induced cytotoxicity in SH‐SY5Y cells [15]. Both equally protected SH‐SY5Y from 6‐OHDA‐induced cell death, indicating that the CEO effect was most likely due to EG [15].

The current study found that 6‐OHDA treatment resulted in a significant drop in the antioxidant enzymes GPx1, GPx4, SOD1, and CAT gene and protein expression levels. Previous research has shown that 6‐OHDA therapy reduces CAT and SOD activities, as well as glutathione levels, in SH‐SY5Y [40, 46]. Furthermore, 6‐OHDA reduced the levels of SOD, CAT, and GPx [46], as well as glutathione [40]. 6‐OHDA causes enhanced oxidative stress by lowering glutathione and SOD activity while increasing NADPH oxidase activity, a ROS‐producing enzyme [47]. The observed changes in GPx in SH‐SY5Y cells further support the idea that GPx plays a role in 6‐OHDA‐induced neurotoxicity and that redox imbalance may be a critical factor [46]. The superoxide radical can interact with NO to form peroxynitrite, an even more toxic molecule that assaults neuronal cells nonspecifically [48]. It has been demonstrated that 6‐OHDA can also produce NO in SH‐SY5Y cells [40].

A comparative examination of the different groups revealed that cells pretreated with CEO had higher expression of these enzymes than the 6‐OHDA group. This improvement is most likely due to the oil’s high EG and other compounds content, together with its strong antioxidant properties. A high intracellular iron concentration combined with a GPx4 deficit, as seen in animal models of PD, results in the accumulation of hydroperoxidized phospholipids (LOOH‐PL). This accumulation causes a ferroptosis mechanism to break down the cell membrane. Strategies such as reducing labile intracellular iron (by deferiprone) or reducing LOOH‐PL (with liproxstatin‐1 or ferrostatin‐1) are promising approaches to inhibiting ferroptosis associated with this disease [49]. Among the GPxs, GPx1 is present in virtually all cell types, while GPx4 is the most dominant glutathione‐dependent peroxidase, known for its ability to prevent membrane LPO and detoxify intracellular lipid ROS ([4]). Overexpression of GPx4 was reported to improve brain defense against ferroptosis, lower lipid peroxide levels, and mitigate pathological damage in an Alzheimer’s animal model [46]. Reducing GPx4 degradation through ubiquitination has become a therapeutic focus for a variety of illnesses, including neurological disorders and cancer [4].

To the best of our knowledge, this is the first study to show a substantial effect of CEO on antioxidant enzymes in the SH‐SY5Y cell line triggered by 6‐OHDA. CEO has been proven to decrease the generation of lipid peroxides in several tissues [50]. CEO’s significant iron chelating activity allowed it to successfully enhance GPx4 expression. Mesole et al. [45] found that EG administration dramatically raised brain GPx levels and tissue SOD activity as compared to the aluminum‐treated group. Moreira et al. [11] found that EG reduced oxidative stress caused by 6‐OHDA in an in vivo model of PD. CEO can increase antioxidant enzyme activity by promoting the production of SOD‐3 or GST‐4, which reduces the formation of ROS in vivo and hence exerts antioxidant effects [51]. EG also displayed Fe3+‐reducing capacity and electron‐donating capabilities, allowing it to neutralize free radicals by producing stable products that exert antioxidant effects [31]. EG effectively inhibits DNA oxidation caused by hydroxyl radicals and LPO, and it has a substantially greater inhibitory effect on hydrogen peroxide than other ROS [52]. CEO also suppresses LPO in erythrocyte membranes, increasing membrane resistance to spontaneous hemolysis and decreasing microviscosity, hence preserving structural integrity and functional activity. CEO significantly reduces the intensity of LPO in the liver and brain of mice [50]. It therefore contributes to scavenge ROS and other free radicals from lipid chains, increases the antioxidant capacity of lipids in these organs, and enhances the activity of antioxidant enzymes in the liver [50]. Clove extract also significantly prevents oxidation‐induced protein damage by reducing the formation of protein carbonyl groups and preventing the loss of protein sulfhydryl groups [53]. β‐Caryophyllene, one of the components of CEO, is also known for its ability to reduce ROS production and promote the recovery of mitochondrial membrane potential (MMP).

This is accomplished by activating nuclear factor erythroid 2 (Nrf2), which protects cells against oxidative stress [54, 55]. Sharma et al. [56] found that clove extract and its principal ingredients had a remarkable capacity to suppress ROS and restore MMP levels. These results suggest an effective protection against oxidative stress induced by H_2_O_2_ on SH‐SY5Y cells due to their antioxidant properties.

## 5. Conclusion

In conclusion, our research demonstrates that CEO has a high yield and a diverse bioactive content. The antioxidant activity of CEO, as proven by DPPH and iron chelation assays, supports its use as a natural alternative to synthetic antioxidants that may induce potential health risks. Our studies on the protective effects of CEO on SH‐SY5Y cells exposed to 6‐OHDA‐induced oxidative stress indicated a significant reduction in DNA damage and enhanced expression of key antioxidant enzymes (GPx1, GPx4, CAT, and SOD1). These findings imply that CEO can protect against oxidative damage, making it a therapeutic strategy option against neurodegenerative diseases like PD.

## Ethics Statement

The experiments followed the animal committee ethical guidelines of Clermont Auvergne University. All the data were performed in a blinded manner so that the experimenter could not recognize the different cell groups and their treatments.

## Consent

All authors consented to participate in this study.

## Conflicts of Interest

The authors declare no conflicts of interest.

## Author Contributions

Conception and design of the study: D.H., A.K., A.H., and C.M.; acquisition and interpretation of data: D.H., C.M., A.H., and A.K.; writing of the article: D.H. and A.H.; revising the article critically for important intellectual content: D.H., A.H., C.M., and A.K.; and final approval of the version to be submitted: D.H., A.K., A.H., O.O., L.O., and C.M.

## Funding

The authors received no specific funding for this work.

## Data Availability

The data that support the findings of this study are available from the corresponding author upon reasonable request.

## References

[1] Migdal C. and Serres M. , Espèces Réactives De L’Oxygène et Stress Oxydant, Médecine/Sciences. (2011) 27, no. 4, 405–412, 10.1051/medsci/2011274405. 21524406

[2] Puspita L. , Chung S. Y. , and Shim J. W. , Oxidative Stress and Cellular Pathologies in Parkinson’s Disease, Molecular Brain. (2017) 10, 1–12, 10.1186/s13041-017-0340-9, 2-s2.0-85035784486. 29183391 PMC5706368

[3] Indo H. P. , Yen H. C. , Nakanishi I. et al., A Mitochondrial Superoxide Theory for Oxidative Stress Diseases and Aging, Journal of Clinical Biochemistry & Nutrition. (2015) 56, 1–7, 10.3164/jcbn.14-42, 2-s2.0-84920453952. 25834301 PMC4306659

[4] Jomova K. , Alomar S. Y. , Alwasel S. H. , Nepovimova E. , Kuca K. , and Valko M. , Several Lines of Antioxidant Defense Against Oxidative Stress: Antioxidant Enzymes, Nanomaterials with Multiple Enzyme-mimicking Activities, and Low-molecular-weight Antioxidants, Archives of Toxicology. (2024) 98, no. 5, 1323–1367, 10.1007/s00204-024-03696-4. 38483584 PMC11303474

[5] Hamer M. A. and Chida Y. , Physical Activity and Risk of Neurodegenerative Disease: A Systematic Review of Prospective Evidence, Psychological Medicine. (2009) 39, no. 1, 3–11, 10.1017/S0033291708003681, 2-s2.0-62249177925. 18570697

[6] Kowalska M. , Piekut T. , Prendecki M. , Sodel A. , Kozubski W. , and Dorszewska J. , Mitochondrial and Nuclear DNA Oxidative Damage in Physiological and Pathological Aging, DNA and Cell Biology. (2020) 39, no. 8, 1410–1420, 10.1089/dna.2019.5347. 32315547

[7] Bender A. , Krishnan K. J. , Morris C. M. et al., High Levels of Mitochondrial DNA Deletions in Substantia Nigra Neurons in Aging and Parkinson Disease, Nature Genetics. (2006) 38, no. 5, 515–517, 10.1038/ng1769, 2-s2.0-33646375711. 16604074

[8] Rodríguez-Arce E. and Saldías M. , Antioxidant Properties of Flavonoid Metal Complexes and Their Potential Inclusion in the Development of Novel Strategies for the Treatment Against Neurodegenerative Diseases, Biomedicine & Pharmacotherapy. (2021) 143, 10.1016/j.biopha.2021.112236. 34649360

[9] Yu M. , Gouvinhas I. , Rocha J. , and Barros I. R. N. A. , Phytochemical and Antioxidant Analysis of Medicinal and Food Plants Towards Bioactive Food and Pharmaceutical Resources, Scientific Reports. (2021) 11, no. 1, 10.1038/s41598-021-89437-4. PMC8113553 33976317

[10] Amir Rawa M. S. , Mazlan M. K. N. , Ahmad R. , Nogawa T. , and Wahab H. A. , Roles of *Syzygium* in Anti-cholinesterase, Anti-diabetic, Anti-inflammatory, and Antioxidant: from Alzheimer’s Perspective, Plants. (2022) 11, no. 11, 10.3390/plants11111476. PMC9183156 35684249

[11] Moreira Vasconcelos C. F. , da Cunha Ferreira N. M. , Hardy Lima Pontes N. et al., Eugenol and its Association with Levodopa in 6-hydroxydopamine-induced Hemiparkinsonian Rats: Behavioural and Neurochemical Alterations, Basic and Clinical Pharmacology and Toxicology. (2020) 127, no. 4, 287–302, 10.1111/bcpt.13425. 32353201

[12] Messaoud C. and Boussaid M. , Myrtus Communis Berry Color Morphs: A Comparative Analysis of Essential Oils, Fatty Acids, Phenolic Compounds, and Antioxidant Activities, Chemistry and Biodiversity. (2011) 8, no. 2, 300–310, 10.1002/cbdv.201000088, 2-s2.0-79951902785. 21337502

[13] Brand-Williams W. , Cuvelier M. E. , and Berset C. L. W. T. , Use of a Free Radical Method to Evaluate Antioxidant Activity, LWT-Food Science and Technology. (1995) 28, no. 1, 25–30, 10.1016/S0023-6438(95)80008-5, 2-s2.0-58149364663.

[14] Yan L. Y. , Teng L. T. , and Jhi T. J. , Antioxidant Properties of Guava Fruits: Comparison with Some Local Fruits, Sunway Acad J. (2006) 3, 9–20.

[15] Hamdi D. , Ouachikh O. , Ouchchane L. , Omara-Reda H. , Messaoud C. , and Hafidi A. , The Neuroprotective Effect of Clove Essential Oil Against 6-OHDA-induced Cell Death in SH-SY5Y and a Rat Model of Parkinson′s disease, Austin Alzheimers J Parkinsons Dis. (2023) 6, no. 2, 10.26420/austinalzheimersjparkinsonsdis.2023.1039.

[16] Batista F. , Vaiman D. , Dausset J. , Fellous M. , and Veitia R. A. , Potential Targets of FOXL2, a Transcription Factor Involved in Craniofacial and Follicular Development, Identified by Transcriptomics, Proceedings of the National Academy of Sciences of the United States of America. (2007) 104, no. 9, 3330–3335, 10.1073/pnas.0611326104, 2-s2.0-33847666428. 17360647 PMC1805535

[17] Kim S. W. , Lee J. H. , Kim B. , Yang G. , and Kim J. U. , Natural Products as the Potential to Improve Alzheimer’s and Parkinson’s Disease, International Journal of Molecular Sciences. (2023) 24, no. 10, 10.3390/ijms24108827. PMC10218422 37240173

[18] Cheesman M. H. , Plants of the Genus *Syzygium* (Myrtaceae): A Review on Ethnobotany, Medicinal Properties and Phytochemistry, Bioactive Compounds of Medicinal Plants: Properties and Potential for Human Health, 2018, Canada: Apple Academic Press., 35–84.

[19] Alfikri F. N. , Pujiarti R. , Wibisono M. G. , and Hardiyanto E. B. , Yield, Quality, and Antioxidant Activity of Clove (*Syzygium aromaticum* L.) Bud Oil at the Different Phenological Stages in Young and Mature Trees, Scientific. (2020) 2020, 1–8, 10.1155/2020/9701701. PMC7290900 32566363

[20] Guan W. , Li S. , Yan R. , Tang S. , and Quan C. , Comparison of Essential Oils of Clove Buds Extracted With Supercritical Carbon Dioxide and Other Three Traditional Extraction Methods, Food Chemistry. (2007) 101, no. 4, 1558–1564, 10.1016/j.foodchem.2006.04.009, 2-s2.0-33947323265.

[21] Nana W. L. , Eke P. , Fokom R. et al., Antimicrobial Activity of *Syzygium aromaticum* and Zanthoxylum xanthoxyloides Essential Oils Against Phytophthora megakarya, Journal of Phytopathology. (2015) 163, no. 7–8, 632–641, 10.1111/jph.12363, 2-s2.0-84935703688.

[22] Selles S. M. A. , Kouidri M. , Belhamiti B. T. , and Ait Amrane A. , Chemical Composition, *in-vitro* Antibacterial and Antioxidant Activities of *Syzygium aromaticum* Essential Oil, Journal of Food Measurement and Characterization. (2020) 14, no. 4, 2352–2358, 10.1007/s11694-020-00482-5.

[23] Ayoola G. A. , Lawore F. M. , Adelowotan T. et al., Chemical Analysis and Antimicrobial Activity of the Essential Oil of *Syzygium aromaticum* (Clove), African Journal of Microbiology Research. (2008) 2, 162–166.

[24] Sokamte T. A. , Jazet D. P. , and Tatsadjieu N. L. , In Vitro Activity of *Syzygium aromaticum* Against Food Spoilage Fungi and its Potential Use as an Antiradical Agent, Journal of Microbiology Research. (2016) 6, no. 1, 1–7.

[25] Lee S. , Najiah M. , Wendy W. , and Nadirah M. , Chemical Composition and Antimicrobial Activity of the Essential Oil of *Syzygium aromaticum* Flower Bud (Clove) Against Fish Systemic Bacteria Isolated From Aquaculture Sites, Frontiers of Agriculture in China. (2009) 3, 332–336, 10.1007/s11703-009-0052-8, 2-s2.0-70349504165.

[26] Alitonou G. A. , Tchobo F. P. , Avlessi F. et al., Chemical and Biological Investigations of *Syzygium aromaticum* L. Essential Oil from Benin, International Journal of Brain and Cognitive Sciences. (2012) 6, no. 3, 1360–1367, 10.4314/ijbcs.v6i3.37.

[27] Barakat H. , Composition, Antioxidant, Antibacterial Activities and Mode of Action of Clove (*Syzygium aromaticum* L.) Buds’ Essential Oil, British Journal of Applied Science & Technology. (2014) 4, no. 13, 1934–1951, 10.9734/bjast/2014/8902.

[28] Saeed A. and Shahwar D. , Evaluation of Biological Activities of the Essential Oil and Major Component of Syzygium aromaticum, Journal of Animal and Plant Sciences. (2015) 25, no. 4, 1095–1099.

[29] Ogata M. , Hoshi M. , Urano S. , and Endo T. , Antioxidant Activity of Eugenol and Related Monomeric and Dimeric Compounds, Chemical and Pharmaceutical Bulletin. (2000) 48, no. 10, 1467–1469, 10.1248/cpb.48.1467, 2-s2.0-0033791895. 11045452

[30] Radünz M. , da Trindade M. L. , Camargo T. M. et al., Antimicrobial and Antioxidant Activity of Unencapsulated and Encapsulated Clove (Syzygium aromaticum, L.) Essential Oil, Food Chemistry. (2019) 276, 180–186, 10.1016/j.foodchem.2018.09.173, 2-s2.0-85054452413. 30409582

[31] Gülçin İI. , Antioxidant Activity of Eugenol: A Structure–activity Relationship Study, Journal of Medicinal Food. (2011) 14, no. 9, 975–985, 10.1089/jmf.2010.0197, 2-s2.0-80051960180. 21554120

[32] Vella F. M. , Calandrelli R. , Cautela D. , Fiume I. , Pocsfalvi G. , and Laratta B. , Chemometric Screening of Fourteen Essential Oils for Their Composition and Biological Properties, Molecules. (2020) 25, no. 21, 10.3390/molecules25215126. PMC7663352 33158110

[33] Zhang K. , Chemical Composition and Antioxidant Activities of the Essential Oil from the Clove Buds (*Syzygium aromaticum*) Toward Various Oxidative Stresses *in Vitro* , Agr Food Sci Res. (2015) 2, no. 1, 19–24.

[34] Kennouche A. , Benkaci-Ali F. , Scholl G. , and Eppe G. , Chemical Composition and Antimicrobial Activity of the Essential Oil of *Eugenia caryophyllata* Cloves Extracted by Conventional and Microwave Techniques, Journal of Biologically Active Products from Nature. (2015) 5, no. 1, 1–11, 10.1080/22311866.2014.961100, 2-s2.0-84952951099.

[35] Gülçin Ì , Güngör Ì , Beydemir S. S. , Elmasta M. , and Küfrevioglu I. , Comparison of Antioxidant Activity of Clove (*Eugenia Caryophylata* Thunb) Buds and Lavender (*Lavandula stoechas* L.), Food Chemistry. (2004) 87, no. 3, 393–400, 10.1016/j.foodchem.2003.12.008, 2-s2.0-2442558300.

[36] Ratan R. R. , Murphy T. H. , and Baraban J. M. , Rapid Communication: Oxidative Stress Induces Apoptosis in Embryonic Cortical Neurons, Journal of Neurochemistry. (1994) 62, no. 1, 376–379, 10.1046/j.1471-4159.1994.62010376.x, 2-s2.0-0028095981. 7903353

[37] Cooke M. S. , Evans M. D. , Dizdaroglu M. , and Lunec J. , Oxidative DNA Damage: Mechanisms, Mutation and Disease, The FASEB Journal. (2003) 17, no. 10, 1195–1214, 10.1096/fj.02-0752rev, 2-s2.0-0038799736. 12832285

[38] Cheng K. C. , Cahill D. S. , Kasai H. , Nishimura S. , and Loeb L. A. , 8-Hydroxyguanine, an Abundant Form of Oxidative DNA Damage, Causes G⟶T and A⟶C Substitutions, Journal of Biological Chemistry. (1992) 267, no. 1, 166–172, 10.1016/s0021-9258(18)48474-8. 1730583

[39] Yasuhara T. , Hara K. , Sethi K. D. , Morgan J. C. , and Borlongan C. V. , Increased 8-OHdG Levels in the Urine, Serum, and Substantia Nigra of Hemiparkinsonian Rats, Brain Research. (2007) 1133, 49–52, 10.1016/j.brainres.2006.11.072, 2-s2.0-33846315383. 17188662

[40] Cirmi S. , Maugeri A. , Lombardo G. E. et al., A flavonoid-rich Extract of Mandarin Juice Counteracts 6-OHDA-induced Oxidative Stress in SH-SY5Y Cells and Modulates Parkinson-related Genes, Antioxidants. (2021) 10, no. 4, 10.3390/antiox10040539. PMC8066648 33808343

[41] Pradhan S. H. , Liu J. Y. , and Sayes C. M. , Evaluating Manganese, Zinc, and Copper Metal Toxicity on SH-SY5Y Cells in Establishing an Idiopathic Parkinson’s Disease Model, International Journal of Molecular Sciences. (2023) 24, no. 22, 10.3390/ijms242216129. PMC10671677 38003318

[42] Kobayashi H. , Oikawa S. , Umemura S. , Hirosawa I. , and Kawanishi S. , Mechanism of Metal-mediated DNA Damage and Apoptosis Induced by 6-hydroxydopamine in Neuroblastoma SH-SY5Y Cells, Free Radical Research. (2008) 42, no. 7, 651–660, 10.1080/10715760802270334, 2-s2.0-48449092397. 18654880

[43] Betarbet R. , Sherer T. B. , and Greenamyre J. T. , Animal Models of Parkinson′s Disease, BioEssays. (2002) 24, no. 4, 308–318, 10.1002/bies.10067, 2-s2.0-0036010592. 11948617

[44] Blum D. , Torch S. , Lambeng N. et al., Molecular Pathways Involved in the Neurotoxicity of 6-OHDA, Dopamine and MPTP: Contribution to the Apoptotic Theory in Parkinson′s Disease, Progress in Neurobiology. (2001) 65, no. 2, 135–172, 10.1016/s0301-0082(01)00003-x, 2-s2.0-0034993599. 11403877

[45] Mesole S. B. , Alfred O. O. , Yusuf U. A. , Lukubi L. , and Ndhlovu D. , Apoptotic Inducement of Neuronal Cells by Aluminium Chloride and the Neuroprotective Effect of Eugenol in Wistar Rats, Oxidative Medicine and Cellular Longevity. (2020) 2020, 1–7, 10.1155/2020/8425643. PMC7008282 32089784

[46] Chen C. H. , Hsu P. C. , Hsu S. W. et al., Protective Effects of Jujubosides on 6-OHDA-induced Neurotoxicity in SH-SY5Y and SK-N-SH Cells, Molecules. (2022) 27, no. 13, 10.3390/molecules27134106. PMC9268520 35807356

[47] Rodriguez‐Pallares J. , Parga J. A. , Munoz A. , Rey P. , Guerra M. J. , and Labandeira‐Garcia J. L. , Mechanism of 6‐hydroxydopamine Neurotoxicity: the Role of NADPH Oxidase and Microglial Activation in 6‐hydroxydopamine‐induced Degeneration of Dopaminergic Neurons, Journal of Neurochemistry. (2007) 103, no. 1, 145–156, 10.1111/j.1471-4159.2007.04699.x, 2-s2.0-34548620779. 17573824

[48] Szabo C. , Ischiropoulos H. , and Radi R. , Peroxynitrite: Biochemistry, Pathophysiology and Development of Therapeutics, Nature Reviews Drug Discovery. (2007) 6, no. 8, 662–680, 10.1038/nrd2222, 2-s2.0-34547673497. 17667957

[49] Moreau C. , Rolland A. S. , Guyon P. et al., Nouvelle Stratégie de Neuroprotection Basée sur la Chélation Conservatrice du fer dans la Maladie de Parkinson, Bulletin de l′Académie Nationale de Médecine. (2019) 203, no. 6, 415–423, 10.1016/j.banm.2019.04.019, 2-s2.0-85071911249.

[50] Misharina T. A. , Fatkullina L. D. , Alinkina E. S. et al., Effects of Low Doses of Essential Oil on the Antioxidant State of the Erythrocytes, Liver, and the Brains of Mice, Prikladnaia biokhimiia i mikrobiologiia. (2014) 50, no. 1, 101–107, 10.7868/s0555109914010097, 2-s2.0-84907605828. 25272759

[51] Zhang L. , Gu B. , and Wang Y. , Clove Essential Oil Confers Antioxidant Activity and Lifespan Extension in *C. elegans* via the DAF-16/FOXO Transcription Factor, Comparative Biochemistry and Physiology-Part C: Toxicology & Pharmacology. (2021) 242, 10.1016/j.cbpc.2020.108938. 33171300

[52] Nam H. and Kim M. M. , Eugenol With Antioxidant Activity Inhibits MMP-9 Related to Metastasis in Human Fibrosarcoma Cells, Food and Chemical Toxicology. (2013) 55, 106–112, 10.1016/j.fct.2012.12.050, 2-s2.0-84873274042. 23313798

[53] Suantawee T. , Wesarachanon K. , Anantsuphasak K. et al., Protein Glycation Inhibitory Activity and Antioxidant Capacity of Clove Extract, Journal of Food Science and Technology. (2015) 52, no. 6, 3843–3850, 10.1007/s13197-014-1452-1, 2-s2.0-84929952131. 26028769 PMC4444878

[54] Assis L. , Straliotto M. R. , Engel D. , Hort M. A. , Dutra R. C. , and De Bem A. F. , β-Caryophyllene Protects the C6 Glioma Cells Against glutamate-induced Excitotoxicity Through the Nrf2 Pathway, Neuroscience. (2014) 279, 220–231, 10.1016/j.neuroscience.2014.08.043, 2-s2.0-84907484851. 25194788

[55] Wang G. , Ma W. , and Du J. , β-Caryophyllene (BCP) Ameliorates MPP+ Induced Cytotoxicity, Biomedicine & Pharmacotherapy. (2018) 103, 1086–1091, 10.1016/j.biopha.2018.03.168, 2-s2.0-85046105091. 29710667

[56] Sharma H. , Kim D. Y. , Shim K. H. , Sharma N. , and An S. S. A. , Multi-Targeting Neuroprotective Effects of Syzygium aromaticum Bud Extracts and Their Key Phytocompounds Against Neurodegenerative Diseases, International Journal of Molecular Sciences. (2023) 24, no. 9, 10.3390/ijms24098148. PMC10178913 37175851

